# Renal safety of tenofovir containing antiretroviral regimen in a Singapore cohort

**DOI:** 10.1186/1742-6405-9-19

**Published:** 2012-06-15

**Authors:** Arlene C Chua, Ryan M Llorin, Kelvin Lai, Philippe Cavailler, Hwa Lin Law

**Affiliations:** 1 Department of Infectious Disease, Tan Tock Seng Hospital, 11 Jalan Tan Tock Seng, Singapore, 308433, Singapore; 2 Department of Pharmacy, Tan Tock Seng Hospital, 11 Jalan, Tan Tock Seng, Singapore, 308433, Singapore; 3 Department of Epidemiology Research, Regional Emerging Diseases Intervention Centre, 10 Biopolis Road #02-01, Singapore, 138670, Singapore

**Keywords:** Tenofovir, Kidney, Antiretrovirals

## Abstract

**Background:**

Tenofovirdisoproxilfumarate (TDF) is a nucleotide analogue widely recommended in international HIV treatment guidelines. The association of TDF and renal dysfunction has remained an area of interest.

**Findings:**

We conducted a retrospective review of all patients on TDF from July 2007 to December 2009 in our institution and evaluated their renal function. Absolute change of creatinine clearance (CL_Cr_) using Cockroft-Gault equation from baseline was calculated at 6, 12, 18 and 24 months. Overall, 226 patients were included in the study. Ninety percent were male. The median age was 46 yrs old (23–82), median weight was 60 kg (IQR 53.75-68), median CD4 count was 127 cells/mm^3^ (IQR 38–258) and median CL_Cr_ 82.7 mL/min (IQR 71.4-101.7) on initiation of TDF. The median decline of CL_Cr_ from baseline was −3.9 ml/min (IQR −12.3 to 7.6), and −3.6 ml/min (IQR −12.4 to 6.7) at 12 (n = 102), 24 months (n = 75) respectively. Eighteen of 226 patients had a decline in renal function to </=50 ml/min. Majority of which had an improvement of CL_Cr_ on follow up. Only 80% of patients ever received monitoring of renal function.

**Conclusion:**

While we noted renal toxicity to be rare and transient among our cohort receiving TDF as part of their ARV regimen, these results reflect the short term renal effects of TDF given that ARV treatment is lifelong. Given that laboratory monitoring may be difficult to implement in many situations, future prospective studies looking into an evidence based algorithm for less frequent renal function monitoring than current guideline recommendations may be helpful.

## Background

Tenofovirdisoproxilfumarate (TDF) is a nucleotide analogue widely recommended in international HIV treatment guidelines [1-3].

The association of TDF and renal dysfunction has remained an area of interest. In several clinical trials, TDF has been found to be safe with renal effects reported to be 1-3% with minimal differences from comparative non TDF arms [4-7]. However, several case reports and series show patients treated with TDF may present with acute renal failure, Fanconi syndrome and diabetes insipidus. These reports suggested that TDF could cause renal tubular injury and decline in creatinine clearance (CL_Cr_) [8-14]. A meta-analysis of prospective studies comparing TDF-containing with non-TDF containing ARV regimens showed that TDF use was associated with statistically significant loss of renal function and the clinical effect was modest [15].

The mechanism by which TDF causes renal toxicity is not well characterized and some studies suggest TDF induces mitochondrial toxicity [16]. Pharmacogenetics has been explored as one potential risk factor [17]. Goicoechea et al have reported treatment with TDF and a protease inhibitor (PI) to be associated with greater declines in renal function compared to TDF with nonnucleoside reverse transcriptase inhibitor (NNRTI) based therapy [18].

Current international guidelines [2] recommend monitoring renal function at ARV initiation or modification, 2–8 weeks post ARV initiation or modification and every 3–6 months thereafter and when clinically indicated. Dose reduction for TDF is recommended when the falls CL_Cr_ below 50 ml/min.

We evaluated our practice of monitoring for renal function in our local cohort of HIV infected individuals receiving TDF in Singapore as well as investigated the changes of renal function over time. We specifically looked at the proportion of patients whose CL_Cr_ fell below 50 ml/min requiring TDF dose adjustment after treatment and subsequent management of these cases.

## Methods

We conducted a retrospective review of all patients who were started on an antiretroviral regimen containing TDF from July 2007 to December 2009 at the Communicable Disease Centre (CDC) at Tan Tock Seng Hospital (TTSH) in Singapore. Data was collected from review of medical records. Data on demographics, ARV, comorbidities and TDF dose were collected. Creatinine clearance (CL_Cr_) was calculated using the Cockcroft-Gault equation.

Absolute change of CL_Cr_ from baseline was calculated for each patient at 3, 6, 9, 12, 15, 18, 21 and 24 months. Not all patients had the same frequency of serum creatinine monitoring because of physician differences in clinical management. When available, the CL_Cr_ at follow up was calculated using the serum creatinine level closest to the period of evaluation (+/− 1 month). Evaluations were made on the associations of other variables with change in CL_Cr_, including age, ethnicity, sex, diabetes, hypertension, hepatitis B coinfection, presence of chronic renal disease, CD4 cell count and NNRTI or PI based regimen.

Monitoring of serum creatinine, appropriateness of dose of TDF based on CL_Cr_, and deaths among those who developed renal toxicity were evaluated.

We defined renal toxicity as a reduction of CL_Cr_ to </= 50 ml/min. It is recommended that the dosing interval of TDF be modified for patients with a CL_Cr_ of <50. Bygrave et al published their experience with TDF defining renal toxicity by a reduction of CL_Cr_ to </= 50 ml/min [19].

For the baseline characteristics we used medians and interquartile ranges (IQRs) for continuous variables while counts and proportions were used for categorical variables. The proportion of creatinine measurements performed at baseline and follow up were calculated. The proportion of patients who received an appropriate TDF dose based on baseline CL_Cr_ was obtained. The median change of CL_Cr_ from baseline was calculated at 3, 6, 9, 12, 15, 18, 21 and 24 months. SPSS for Windows version 18.0 (SPSS Inc. Chicago, IL, USA) was used for all statistical analysis. All tests were two tailed. In all tests, a p value <0.05 was considered significant. We compared proportions using Fisher’s exact test, means using ANOVA test and medians using Kruskal Wallis test.

This study was reviewed and approved by the Singapore National Health Care Group (NHG) Domain Specific Ethics Review Board Committee.

## Results

From July 2007 to December 2009, there were a total of 226 patients who received an antiretroviral regimen containing TDF. Baseline demographic and clinical characteristics of patients receiving TDF are presented in Table 1.

**Table 1:** Characteristics of patients who received Tenofovir, TTSH, Singapore, Jul. 2007 – Dec. 2009 (N = 226)

	**No (%) of patients at TDF Initiation**
*Sex*	
Male	203 (89.9)
Female	23 (10.1)
*Ethnicity*	
Chinese	176 (78.6)
Malay	26 (11.6)
Indian	10 (4.5)
Others	12 (5.3)
*Age*	
Median (range)	46 (23 – 82)
*Weight*	
Median (IQR)	60.0 [53.75 - 68.0]
*ART Regimen*	
NNRTI-based	118 (52.2)
PI - based	108 (47.8)
*Co-morbidity*	
Hepatitis B co-infection	63 (27.9)
Diabetes mellitus	26 (11.5)
Hypertension	23 (10.2)
Chronic renal disease	2 (0.9)
*CD4 cell count (cells/mm*^*3*^*)*	
Median [IQR]	127 [38–258]
*Viral Load (Log*_*10*_*Copies/mL)*	
Median [IQR]	4.9 [4.2-5.5]
*Creatinine clearance (mL/min)*	
Median [IQR]	82.7 [71.4-101.7]
>/= 90	72 (43.3)
60-89	73 (43.9)
30-59	20 (12.2)
<30	1 (0.6)

A total of 16 patients had discontinuation of TDF. The reasons for stopping therapy included virological failure (n = 11), change of TDF to another NRTI due to patient preference unrelated to TDF toxicity (n = 3) and renal toxicity (n = 2).

There were 166 (73.5%) patients with a baseline creatinine measured at initiation of TDF and the median CL_Cr_ was 82.7 mL/min (IQR 71.4-101.7). Subsequent serum creatinine was obtained in 192 (84.9%) patients within 6 months and 202 (89.4%) within 12 months of starting TDF. The patients did not have the same frequency of serum creatinine measurements during the follow up period. Creatinine has been checked for only 53% (102/194) and 77% (75/98) for patients in follow up at 12 and 24 month respectively.

Of those patients who had a baseline serum creatinine at initiation, 12 patients had a calculated CL_Cr_ of <50 mL/min. Among these, 11/12 (92%) had an incorrect TDF dose. The dose of TDF was adjusted for 5 patients on the following visit, and for 3 patients, repeat CL_Cr_ was >50 mL/min requiring no further TDF dose adjustment.

**Figure 1:**
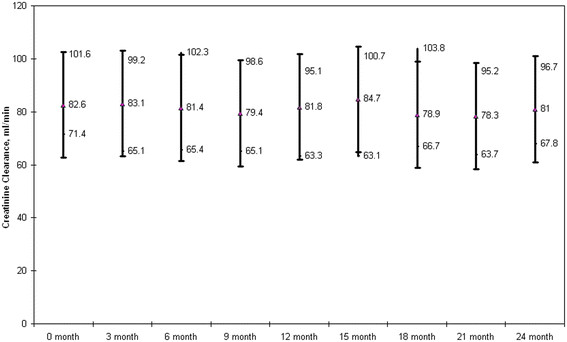
Median Creatinine Clearance over time.

The median decline of CL_Cr_ from baseline of CL_Cr_ was −3.9 ml/min (IQR −12.3 to 7.6) and −3.6 ml/min (IQR −12.4 to 6.7) at 12 (n = 102) and 24 months (n = 75) respectively (Figure 1). There was no statistical association between the trend in CL_Cr_ and the CD4 count,, age, NNRTI or PI based ARV regimen, or the presence of an existing co-morbidity (Hepatitis B coinfection, hypertension or diabetes).

There were 18 patients who had a baseline CL_Cr_ of >50 mL/min who developed toxicity on follow up with a subsequent CL_Cr_ of</= 50 mL/min. The onset of CL_Cr_ decline to </=50 mL/min in 10/18 patients was within 6 months of TDF initiation. The CL_Cr_ decline was >25% for 16 patients and >50% for 2 patients. Only 1 patient developed severe renal impairment (CL_Cr_ <30 ml/min). There were 3/18 that died of which none were attributed to TDF toxicity. Of the 15 remaining patients, 2 were switched to a non TDF containing regimen and 13 patients remained on TDF despite renal impairment. The CL_Cr_ improved without dose adjustment in the majority of these patients (10 of 13). The remaining 3/10 patients had TDF dose adjustment to 300 mg once every other day based on reduced CL_Cr._

We compared these 18 patients with those who did not have any renal toxicity and found no differences in terms of their baseline CD4 count, age, gender, presence of hypertension, diabetes, or hepatitis B status. There were 12/18 patients who were on a PI based regimen. There was a tendency for reduction of Cl_Cr_ among those who had a PI based regimen (p = 0.38, at 3 months) however this was not significant.

## Discussion

Our study showed that in an Asian HIV population, majority of whom had a low CD4 count but relatively normal CL_Cr_, the proportion of patients who develop renal toxicity is low (2.2%; 5/226). While use of protease inhibitor has been associated with greater decline in renal function [18], we are unable to show any significant difference which may be attributed to the small number in our study.

In our clinical practice setting around 80% of patients on TDF ever received monitoring of renal function.

The data on TDF related renal toxicity in an Asian HIV cohort is limited. A recent observational Japanese cohort study showed that the incidence of renal dysfunction in low body weight patients treated with tenofovir was twice as high compared to those treated with abacavir [20]. A study looking at the incidence and predictor of TDF associated renal toxicity in Thai patients is currently ongoing [21].

One limitation of our study is that we calculated CL_Cr_ using the Cockroft-Gault equation. Studies have shown that the abbreviated MDRD equation performs better than the Cockroft Gault equation for non Caucasian ethnic groups [22-24].

Other limitations of our study are that we did not have a non TDF comparator group. Our follow up period is limited to up to 24 months which only 98 out of 226 patients have achieved. It would be important to see CL_Cr_ trend over longer periods as other studies have shown decline in renal function long term [25,26].

Limitations of using retrospective data obtained from medical records include incomplete data, difficulty in verifying documented information, and variability in the quality of documentation among physicians. Moreover, not all patients had creatinine measured during the designated follow up period due to physician variability in clinical practice. Our study cohort includes both ARV naïve and experienced patients which makes our results difficult to compare with other studies.

Our study showed that renal toxicity was uncommon and transient which confirms previous studies on TDF. Given that ARV therapy is lifelong, the results of our study reflect the short term renal effects of TDF. Current international guidelines recommend monitoring renal function at ARV initiation or modification, 2–8 weeks and every 3–6 months thereafter [2]. Given that laboratory monitoring may be difficult to implement in many situations, future prospective studies looking into an evidence based algorithm for less frequent renal function monitoring than current guideline recommendations may be helpful.

## Competing interests

All authors declare that they have no competing interests.

## Author’s contributions

AC wrote the manuscript, designed the study and analyzed the data. RL participated in the collection of data. PC participated in the collection of data and analyzed the data. KL participated in the collection of data. HL participated in the design of the study. All authors have read and approved the final manuscript.
